# Oncologist and General Practitioner Perspectives of Telehealth‐Delivered Colorectal Cancer Survivorship Care

**DOI:** 10.1002/pon.70491

**Published:** 2026-05-19

**Authors:** Karolina Lisy, Matthew Tieu, Erin Laing, Claire Gore, Penelope Schofield, Raymond J. Chan, Jon Emery, Andrew Martin, Richard De Abreu Lourenco, Michael Jefford

**Affiliations:** ^1^ Centre for Health Services Research in Cancer Peter MacCallum Cancer Centre Melbourne Victoria Australia; ^2^ Australian Cancer Survivorship Centre Peter MacCallum Cancer Centre Melbourne Victoria Australia; ^3^ Sir Peter MacCallum Department of Oncology University of Melbourne Parkville Victoria Australia; ^4^ School of Public Health Adelaide University Adelaide South Australia Australia; ^5^ Nutrition & Speech Pathology Department Peter MacCallum Cancer Centre Melbourne Victoria Australia; ^6^ School of Exercise & Nutrition Sciences Deakin University Burwood Victoria Australia; ^7^ Psychosocial Oncology Program Peter MacCallum Cancer Centre Melbourne Victoria Australia; ^8^ School of Computing, Engineering and Mathematical Sciences La Trobe University Melbourne Australia; ^9^ Department of Psychology Swinburne University of Technology Melbourne Victoria Australia; ^10^ Iverson Health Innovation Research Institute Swinburne University of Technology Melbourne Australia; ^11^ Caring Futures Institute Flinders University Adelaide Australia; ^12^ Centre for Cancer Prevention and Early Detection Lee Kong Chian School of Medicine Nanyang Technological University Singapore Singapore; ^13^ Department of General Practice and Primary Care University of Melbourne Melbourne Victoria Australia; ^14^ The University of Queensland Clinical Trial Capability (ULTRA) Centre for Clinical Research University of Queensland Brisbane Queensland Australia; ^15^ Centre for Health Economics Research and Evaluation Faculty of Health University of Technology Sydney Sydney New South Wales Australia

**Keywords:** cancer survivors, colorectal cancer, follow‐up care, models of care, oncology, primary care, survivorship, survivorship care, telehealth

## Abstract

**Background:**

Telehealth for follow‐up of survivors of colorectal cancer (CRC) represents an opportunity to reduce health resource use and provide flexibility for patients and healthcare providers (HCPs). Understanding the perspectives of HCPs who have delivered telehealth‐based CRC survivorship care is critical in determining whether this is an acceptable and appropriate model.

**Aims:**

To examine the experiences of oncologists and general practitioners (GPs) who provided telehealth for CRC survivors within the Shared Care for Colorectal Cancer (SCORE) trial, focusing on implementation outcomes and perceived facilitators and barriers to implementation.

**Methods:**

This qualitative descriptive study involved semi‐structured interviews. Interviews were audio‐recorded and transcribed, and data analyzed using inductive thematic analysis.

**Results:**

Twenty‐three HCPs (13 GPs, 10 oncologists) participated. Seven themes were identified: (1) forced adoption and rapid implementation of telehealth during COVID; (2) patient benefits of convenience and safety; (3) patient factors that determine appropriateness for telehealth; (4) clinical factors that preclude telehealth; (5) HCP preferences and views on quality of care; (6) technical and administrative barriers; and (7) training, technology, administrative support and reimbursement to support future telehealth use. Telehealth was considered acceptable for routine CRC follow‐up care, however required judicious use. Challenges were identified regarding technology, logistical and administrative processes, and the potential for disparities in telehealth access.

**Conclusions:**

This study supports the use of telehealth for the provision of CRC survivorship care, provided that certain patient‐related and clinical characteristics are met. Future research may address decision making for telehealth use and understanding and mitigating disparities in access.

**Trial Registration:**

The Shared Care for Colorectal Cancer (SCORE) Trial is registered with the Australian New Zealand Clinical Trials Registry, ACTRN12617000004369p. Registered on 3 January 2017; protocol version 4 approved 24 February 2017

## 1 Background

Colorectal cancer (CRC) is one of the most prevalent cancers in Australia and worldwide [1]. With advances in screening, detection and treatment, survival rates for people with CRC are improving, with a 5 year survival rate of 72% in Australia [2] and 65% in the United States [3]. Survivors of CRC may face a range of physical, psychological or social concerns related to their cancer or treatment that may persist for many years beyond treatment and negatively impact quality of life [4]. Traditionally, survivorship care for CRC has been oncologist‐led and based in hospitals or cancer centers, however this model is expensive and not sustainable given the growing number of CRC survivors [5, 6]. The specialist‐led model, which tends to focus on surveillance for recurrence [7], may not adequately provide survivors with the full breadth of recommended survivorship care, particularly regarding psychosocial care, lifestyle modification and comorbidity care—these elements may be better situated in primary care [8].

For these reasons, there has been international interest in alternative models of survivorship care, including general practitioner (GP)‐led care, shared specialist and GP care, and nurse‐led care, with a number of trials demonstrating that these alternative models lead to equivalent outcomes and lower costs than traditional specialist‐led care [6, 9]. Most recently, the shared care for colorectal cancer survivors (SCORE) randomized controlled trial (RCT) completed, demonstrating that shared care resulted in non‐inferior quality of life outcomes for survivors, lower costs to the health care system, and was preferred by patients, relative to specialist‐led care [10].

The COVID‐19 pandemic resulted in changes in health care processes and access due to social distancing and quarantine requirements throughout much of the world. There was a surge in the use of telecommunication technologies to provide health care, or telehealth, across many health services, including survivorship care. The SCORE trial completed in May 2021, and as such was affected by the COVID‐19 pandemic [10]. Within SCORE, many appointments in both specialist‐led and shared care arms of the trial were conducted via telehealth; thus an opportunity emerged to investigate telehealth as an acceptable means of delivering survivorship care for CRC survivors.

Though telehealth is not new, prior to the COVID‐19 pandemic its use in survivorship care had been limited and not well studied. To‐date, literature on the use of telehealth to facilitate survivorship care has focused primarily on breast cancer, or mixed cancer populations [11]. In these groups the use of telehealth or telemedicine to aid surveillance and management of the physical and psychosocial effects of cancer, as well as health promotion and disease prevention, has been shown as effective [11]. However, evidence regarding telehealth use for other aspects of recommended survivorship care, including surveillance of recurrent or new cancers and chronic disease management, remains limited [11]. Both patient and physician satisfaction with telehealth consultation as an alternative to in‐person consultation during COVID‐19 has been demonstrated in populations undergoing treatment for CRC [12, 13]. However, no studies address the health care provider (HCP) perspective and experience of using telehealth for providing recommended CRC follow‐up care. An overview of systematic reviews that identified a substantial body of evidence to support the use of telehealth in post‐treatment care also demonstrated a critical lack of evidence addressing the use of telehealth for surveillance for recurrent or new cancers [11].

The objective of this study was to examine the experiences and perspectives of HCPs (both oncologists and GPs) who provided follow‐up care via telehealth during the COVID‐19 pandemic as part of the SCORE RCT, examining the adoption, acceptability, appropriateness and sustainability of delivering survivorship care to CRC survivors via telehealth, as well as facilitators and barriers to its use.

## 2 Methods

### 2.1 Design

This study followed qualitative descriptive methodology underpinned by constructivist principles [14], and was informed by the outcomes for implementation research defined by Proctor et al. [15] Consolidated criteria for reporting qualitative data (COREQ) [16] were followed.

### 2.2 Setting

This study is a substudy of the SCORE RCT, with the full study described elsewhere [10, 17]. Briefly, CRC survivors recruited from five public hospitals were randomized to usual care (follow‐up care with their oncologist according to standard practice) or to shared care arms. For shared care, two hospital appointments were replaced with GP visits at three and nine months post‐treatment completion, with an additional GP visit soon after treatment completion. Patients in the shared care arm also received a survivorship care plan (SCP; shared with the GP and specialist), patient information booklet and DVD, and a common issues and concerns checklist, and GPs received clinical management guidelines. SCORE was conducted over two stages between 24 February 2017 and 27 August 2018 (stage 1) and 18 October 2019 and 28 May 2021 (stage 2) due to sequential research funding. Due to COVID‐19, follow‐up care during stage 2 was largely delivered by telehealth from March 2021 to trial completion, and questions regarding delivery of CRC follow‐up care using telehealth were added to the planned implementation evaluation (reported on here [18]). The Peter MacCallum Cancer Center Human Research Ethics Committee granted ethical approval to conduct the study (Ref: HREC/72311/PMCC‐2020).

### 2.3 Participants

Eligible participants were HCPs, both oncologists and GPs, who provided care for CRC survivors within the SCORE RCT. Participants were recruited via email (specialists) and both email and letter (GPs), with non‐responders followed up by phone call. Emails/letters contained information about the study and contact details for the study team. Incentives for GPs ($100 retail gift cards) were offered. Recruitment continued until researchers (MT and KL) agreed that no new data were being expressed in interviews and data saturation [19] was being reached. This occurred after 20 (11 GP and 9 specialist) interviews had been completed; interviews that had already been scheduled proceeded, but no new participants were recruited from this point. Each participant received a plain language summary of study details including study purpose, methods, confidentiality procedures, ethical approval and a contact for further questions, and each participant provided informed consent, which was audio recorded, prior to interview.

### 2.4 Data Collection

One researcher (MT) conducted semi‐structured interviews via telephone or video call (Zoom or MS Teams) from November 2022 to April 2023. MT is a research fellow, and an experienced qualitative researcher and interviewer. Interview schedules were created based on HCP‐level implementation outcomes (acceptability, adoption, appropriateness) for implementation research defined by Proctor et al. [15] (Supporting Information S1: Appendix 1) Interviews aimed to understand how HCPs used telehealth for CRC follow‐up care during the COVID‐19 pandemic (adoption), perspectives regarding acceptability and appropriateness of providing CRC follow‐up care by telehealth, as well as providers' overall experiences, perceptions of facilitators and barriers to providing follow‐up care via telehealth, and sustainability of remote follow‐up. Interviews were recorded and transcribed verbatim using a professional transcription service. Transcripts were not returned to participants in order to minimize the time burden of study participation.

### 2.5 Data Analysis

Transcripts were imported into NVivo (QSR International Pty Ltd). Two experienced qualitative researchers (KL and CG) analyzed the data using inductive thematic analysis, following Braun and Clarke's six‐phase approach [20]. One researcher (KL) closely read and re‐read all transcripts and applied descriptive codes to the text, where the codes were labels that characterized the meaning of each interview segment. A second researcher (CG) independently co‐coded a subset of transcripts (10%) to establish intercoder reliability [21]. Both researchers then discussed this initial analysis in detail to clarify any inconsistencies in coding and establish mutual agreement on the coding framework being developed. Codes were analyzed to explore patterns and similarities in meaning, then grouped together to develop initial categories (sub themes) and themes. Themes and subthemes were reviewed and refined, and both coders reviewed and agreed on all final themes and subthemes. Final themes and sub themes were named to capture and describe the included data.

## 3 Results

Overall, 27 oncology specialists and 28 GPs were invited to participate, with 10 specialists (SP; 7 medical oncologists and 3 surgeons) and 13 GPs ultimately interviewed. The main reasons for non‐participation were not responding to the invitation or being too busy to participate. Interview transcripts were analyzed and seven themes developed from the data (described below and in Table 1).

**TABLE 1:** Summary of themes, subthemes and illustrative quotes.

Themes and subthemes	Participant quotes
1. Forced adoption and rapid implementation	
Limited telehealth follow‐up prior to COVID‐19	“Only occasionally, for regional patients.” SP10
Lack of reimbursement	“No, you weren't allowed to do it. Not if you want to be reimbursed.” GP4 “There was no item number for telehealth prior to COVID… so all consultations then were face‐to‐face.” SP9
Rapid implementation	“It evolved during COVID. There was a period of time where… it was virtually almost exclusively telehealth.” GP8
2. Telehealth allows for patient convenience and safety	
Infection control and safety	“Sometimes people will say ‘oh, I don't have to wait in a waiting room with all these sick patients’; sometimes people perceive it to be safer for them.” SP6
Patient convenience and access	“The patients love it because they don't have to travel.” GP2 “It's more convenient and less stressful for the patient, it saves them money, it saves them time.” SP1
3. Patient characteristics and preferences determine eligibility for telehealth	
Not for new patients	“For patients were known to me and were familiar to me, there was not a huge difference in terms of picking up the phone to make a call or seem them on video. It is very difficult when you've never met the patient before or haven't seen them many times before.” SP2
Able to use technology, have access to reliable internet	“One would be obviously what the patient is comfortable with, with regards to IT; do they know how to work the video health.” SP6 “Occasionally the wifi on each end—Usually the patient's end, I think—Is not very strong to telehealth may not go ahead or end up having to do phone; probably one in three cases are like that.” SP10
Perceptions of older patients	“It relies on the patient having some comfort and familiarity with using the internet, so for older patients, sometimes that meant that their family members or children had to set it up for them.” SP2
English language and hearing	“You get a sense some people either maybe struggle a little bit with a phone due hearing or comprehension and then also if there was a language barrier.” SP5
Patient preference	“If the patient likes it, we'll continue to do it.” SP10
4. Clinical context and purpose of the appointment guide decision‐making	
Suitable for routine care, well patients	“If you're just discussing results etcetera, yes, I think you can. But I think, you know, some things, the longer discussions…sometimes they're better face‐to‐face.” GP4 “If it routine and it's going to be either good news or look, this is everything sort of fairly clear and planned here, then I found it really good. I found it less—I found it more difficult I guess if I was—If we were worried about that, you know, their blood tests were a bit off or there was a sign of recurrence on a scan; I found delivering that kind of information over telehealth or telephone more difficult because you can't be as empathetic.” SP3
Physical examination limitations	“So follow up, I think it's absolutely fine doing it by telehealth unless there's something specifically you need to assess. For example, if you think they've got incisional hernia, in which case you do need to physically see them.” SP4
Not suitable to deliver bad news	“And then there's obviously the—I guess the medical reason that they're being seen so, you know, if it's a routine, straightforward review, I think video health or telephone health is reasonable but if it's breaking bad news or difficult communication or a complex consultation.” SP6
Triaging patients	“In the clinics that I attend now, I actually pre‐triage them the week beforehand to work out who is most appropriate for face‐to‐face video versus telephone.” SP6
5. Quality of care and limitations of telehealth	
Telehealth is acceptable for follow‐up	“I think it's good with the right person, definitely, yes.” GP4 “Now as long as it’s used judiciously, I think it's excellent.” GP8
Quality of care debates	“I would much prefer face‐to‐face than phone consults… Because part of that assessment, most of the times, is a physical examination.” GP12
Loss clinical acumen and non‐verbal cues	“You can always sort of get more clues from seeing a patient face‐to‐face; you can see how they're sort of…if they're emaciated or something like that.” GP10 “There's a lot of stuff you pick up just by talking to the patient… that you don't pick up just by a phone call or telehealth.” GP6
Rapport, connection and holistic care	“It's probably not the actual clinical care and clinical issues, it's the—all those sort of non‐verbal things that go with it and the empathy side of things that you struggle… There's less sort of chat and getting to know you and it being a bit more of a real person.” SP3 “What tends to happen I think is it becomes a lot more clinical and results are good; any problems, off you go. Whereas I think an in‐person interaction, there's a lot more…holistic care. So sort of discussion around bigger issues and how you're going, you sort of talk about the family, the football; there's a bit more of a human element to the interaction rather than just these are the blood test results and no major problems, next visit sort of—so I guess there's a change in the dynamic there.” SP7
6. Technical and administrative challenges	
Technology failures and connectivity issues	“Calls and stuff were dropping out…it was really difficult.” GP2 “So if patients were living in places where they didn't have particularly good internet connections or they were trying to do it on their mobile phone over 3G, it was terrible.” SP3
Administrative burden and workflow disruption	“And there's no way of doing things because I had no way of ordering electronic like records, blood tests or anything like that. I had to actually ring the resident on the ground to fill in a physical paper slip to be mailed to the patient.” SP8 “There's probably more work from the doctor's point of view because you then have to—you can't just give the patient the blood form and book the scan straight away, you have to do a lot of behind the scenes work.” SP10
Process for payments	“If they are on telehealth… I tell the patient okay, you need to ring up the clinic again to pay; it's just a lot of admin stuff. It's embarrassing; you're on the Zoom, then you need to get the patient to call again to charge the patient.” GP13
Efficiency—gains and challenges	“It's quick. If the patients goes on a lot, you can sort of type your notes as they're talking, which is a bit hard to do in face‐to‐face. You can actually do other things when you're talking. That's the biggest advantage of telehealth.” SP8 “There's often a lot more work if they do identify an issue I need to sort out; I can't examine them, I can't give them blood form.” SP10
7. Training, IT infrastructure, administrative support and reimbursement will support future use	
Continued use	“We continue to offer telephone consults so I'd say yes.” GP7 “Yeah, I think it's been valuable and so I support as long as the hospital keeps supporting it, we would like to keep doing it.” SP5
Hybrid use	“We currently are offering video health where the video health platform is available. Telephone is still available for people that can't do video and don't want to come in face‐to‐face that are safe to do so, otherwise we're very happy with face‐to‐face. So I'm currently doing a hybrid… we just have to work out the balance of things financially based on the best patient care.” SP6
Practitioner training	“I'm quite comfortable—A lot of other GPs are not [doing telehealth’ though because it's a new concept to them and especially if they're risk‐averse; they don't like doing telephone consultations, they'd rather see somebody face‐to‐face. And it's sometimes a matter of just offering those clinicians training in how to conduct a safe and effective telephone or a telehealth consultation.” GP9
Need for IT support	“I think having an IT person to call if there's an issue would be helpful… there's really not a lot of IT support in the clinic if something goes wrong.” SP10
Need for administrative support	“Administrative support is always important in all clinics and domains, particularly when you're looking at multiple forms of technology and multiple apps or platforms which it's done.” SP6
Required IT infrastructure	“I've only worked at corporate practices so corporate practices, a lot of them sometimes they don't feel that it’s cost‐effective to, you know, kit out all computers, [and] the web cams.” GP9 “Available, accessible, stable platform.” SP4
Adequate reimbursement	“I just think that if telehealth is not rebateable for non‐regional patients, it will be a problem; it will be discouraged to use… and also I think phone should also be allowed to have a—Medicare should have an item number… so yeah, so I think it would be beneficial and certainly would encourage people to use it if it's reimbursed.” SP10

### 3.1 Forced Adoption and Rapid Implementation

Telehealth adoption for cancer follow‐up care was overwhelmingly driven by COVID‐19 necessity rather than pre‐existing practice, according to both GPs and specialists. Participants described limited use of telehealth for survivorship care prior to the COVID‐19 pandemic, indicating that telehealth was offered “very, very selectively. And very, very rarely for follow‐up” (SP3). A major reason for this was the lack of Medicare billing codes (numerical codes used for government reimbursement). (“No, we didn't actually because there wasn't a specific Medicare number so no, we didn't offer that, no.” GP9).

In the early stages of the COVID‐19 pandemic, cancer follow‐up care largely shifted to telehealth (“Telehealth only started with COVID, right.” SP10), which was perceived as a positive consequence of the pandemic (“I believe that telehealth consultations are… certainly a gain for us coming out of this sort of pandemic, lockdowns and so on, so I think that's a good thing. That's something good that came out of this.” GP12).

### 3.2 Telehealth Allows for Patient Convenience and Safety

The benefits of telehealth for patients were consistently recognized by both GPs and specialists. Perceived benefits for telehealth included convenience for patients, such as reduced travel and waiting times (“If it's a telehealth, that will save [the patient] traveling, waiting, and it's convenient enough for me.” GP13) and greater access, particularly for patients residing regionally (“There'll be a lot of positives, particularly for people that live a long way away and, you know, they'll find it a lot easier to not travel big distances.” SP3). These benefits were particularly emphasized in the context of patients who are otherwise well (“I think if you're very well and fit and you're fine and your blood test is fine and your scan is fine, it's fantastic because… you're not driving in, grappling to find a park, paying for petrol, sitting for hours in the waiting room, to then come in, sit down and be told that you're fine and well and you just go home again.” SP9).

Patient safety was a further benefit of telehealth indicated by both GPs and specialists, as patients were able to avoid busy waiting rooms and potential exposure to contagions (“The waiting room, you know, may have been full of people with coughs and colds and, you know, cancer patients usually have low immunity; you don't really want them in the waiting room.” GP9).

### 3.3 Patient Characteristics and Preferences Determine Eligibility for Telehealth

Various patient factors were discussed as determining whether telehealth was appropriate or not. These included seeing a patient for the first time, and patient characteristics including ability to access and use technology, English language, hearing ability and patient preference. Being familiar with the patient was important to specialists, who stated that it was not appropriate to see a new patient for follow‐up for the first time over telehealth (“I think it's wrong to see someone for the very first time as video telehealth; you kind of need to see someone, you need to examine them at least that first time.” SP1).

On a practical level, patients needed to have access to the appropriate technology, have reliable internet services, and also be able to navigate the required communication platforms (“I think probably people that you get a sense that they would be able to use the technology.” SP5), and it was suggested that this may be more challenging for older patients (“Telehealth is complicated to set up, particularly if they're older patients.” GP6). To receive adequate care via telehealth, a patient needed to have the ability to communicate health concerns which required health literacy, adequate hearing, and adequate English language skills (“Not just IT literacy but health literacy as well, so safety, you know, you have some patients where you're concerned about their capacity or ability to report things over the phone or over video which, if you see them face‐to‐face there's always the clinical acumen of you look at a patient and you say you are not well.” SP6).

Language barriers and coordinating an interpreter emerged as a challenge that complicated telehealth delivery for culturally and linguistically diverse patient populations. Multiple providers identified language as a key criterion for determining telehealth appropriateness (“The ones that don't work well for telehealth are where you need an interpreter; interpreter interactions work much better in the rooms so that's one where you definitely want the patient in the room.” SP7). While some specialists described workarounds such as “three‐way reviews where you can add an interpreter into the call” (SP2), these often introduced technical or audio quality issues where “no one can understand anyone.” (SP8).

Finally, patient preference was a consideration, with both GPs and specialists reporting that patients generally preferred telehealth to in‐person appointments (“most patients say that they prefer it.” SP1). Some providers framed telehealth as a patient‐centered choice (“I often ask patients next time they come in would they like a face‐to‐face consultation or would they like a telephone?” SP9), while maintaining professional judgment about when patient preference for telehealth must be overridden by necessity for in‐person assessment.

### 3.4 Clinical Context and Purpose of the Appointment Guide Decision‐Making

In addition to individual patient characteristics, telehealth was considered suitable or not depending on the reason for the appointment. Both GPs and specialists indicated that telehealth was suitable for administrative or routine follow‐up appointments (“For results I'll do that. And for certain medication… just a repeat of scripts and blood tests, I will do telehealth.” GP13; “Routine assessment, checking blood test, checking CT, telehealth's absolutely fine.” SP5).

The nature of the appointment mattered significantly to the appropriateness of offering telehealth; addressing or discussing concerning symptoms or results that may indicate recurrence, managing treatment side effects that required physical examination, and delivering bad news were all identified as situations that precluded remote review (“If I were worried… if there was a sign of recurrence on a scan, I found delivering that kind of information over telehealth or telephone more difficult.” SP3). Some HCPs described processes they developed to triage patients prior to their appointment to determine those appropriate for telehealth and those requiring a face‐to‐face appointment.

### 3.5 Quality of Care and Limitations of Telehealth

Most HCPs reported positive views regarding the use of telehealth for providing follow‐up cancer care (“I think that it’s definitely a tool that I am familiar using, am happy to use and I am happy to have it as an adjunct to the normal consultations.” SP2). Providers however held divergent views on whether telehealth could deliver care equivalent to in‐person consultations, and the quality of care was seen as highly context dependent. In keeping with the theme regarding clinical appropriateness, some HCPs viewed the quality of care delivered via telehealth as equivalent to face‐to‐face for “the appropriate clinical presentation” (GP9), some viewed it as better due to the benefits for the patient (“It's better because it's more convenient and less stressful for the patient.” SP1), whereas others indicated that telehealth cannot provide the same quality of care (“Always better in person. Always better.” GP6).

Reasons given for preferring face‐to‐face appointments included the inability to conduct physical examinations using telehealth (“You can't examine anybody over video.” SP3), though it was also acknowledged that “if there was anything I was concerned about where I thought I had to see a person in for an exam, I'd always call them in.” (GP7). Others were concerned with loss of clinical acumen and picking up non‐verbal cues (“You can't examine them over the telephone or look at a rash or watch them get up from the chair and see they're struggling and see that they've lost weight and seeing that they're really not quite right.” SP9). Specialists further identified loss of holistic, relationship‐centered care (“There's less sort of chat and getting to know you and it being a bit more of a real person as opposed to, like it becomes very much a… matter of fact kind of interaction, has been my experience with it.” SP3).

### 3.6 Technical and Administrative Barriers to Telehealth Delivery

Both provider groups experienced substantial technological and administrative barriers that undermined telehealth effectiveness and efficiency. The most commonly reported barrier to telehealth for cancer follow‐up care was technology, at both the patient and health service sides (“I think the key for telehealth to be successful is that they do need to have a reliable internet connection; there have been many times when the video drops out or there's a lag and we have to switch to phone.” SP2). Participants also reported that health services' IT systems were not equipped to facilitate telehealth (“I found that the systems were really not quite up to it. And there's no way of doing things because I had no way of ordering electronic like records, blood tests or anything like that. I had to actually ring the resident on the ground to fill in a physical paper slip to be mailed to the patient.” SP8). GPs also reported the cumbersome nature of organizing payment for telehealth consultations (“Because your patient comes in, we can easily charge them private fees for non‐pensioners. If they are on telehealth, we still can charge them… I tell the patient okay, you need to ring up the clinic again to pay; it's just a lot of admin stuff. It's embarrassing; you're on the Zoom, then you need to get the patient to call again to charge the patient.” GP13).

There were diverse views regarding the efficiency of telehealth, with some participants reporting time savings and others indicating that telehealth led to increases in workload, largely around increased administrative burden (“There's often a lot more work if they do identify an issue I need to sort out; I can't examine them, I can't give them blood form… there's probably more work from the doctor's point of view because you then have to—you can't just give the patient the blood form and book the scan straight away, you have to do a lot of behind the scenes work.” SP10).

### 3.7 Training, IT Infrastructure, Administrative Support and Reimbursement Will Support Future Use

Both GPs and specialists expressed commitment to continued telehealth use for cancer follow‐up care, in the context of selective and judicious use based on consultation purpose, patient characteristics, and clinical context. A hybrid model of care was suggested, where some in‐person appointments are replaced by telehealth where appropriate (“Not for all consultations but I think that it’s definitely a tool that I am familiar using, am happy to use and I am happy to have it as an adjunct to the normal consultations.” SP2). This may be supported by training; some HCPs reported receiving training on how to use telehealth, with others suggesting training may be offered to enable effective use of telehealth (“I'm from the UK originally so we used to do telehealth and telephone consultations quite a lot and I'm quite comfortable—a lot of other GPs are not though because it's a new concept to them and especially if they're risk‐averse… And it's sometimes a matter of just offering those clinicians training in how to conduct a safe and effective telephone or a telehealth consultation.” GP9).

To support sustainability of telehealth, the most common issue that participants cited was the need for adequate technology and IT support (“You need the technology, obviously, very good technology, especially for video calls.” GP4; “I think the first would be some secure video conferencing like hardware, that would help me.” GP2). Required infrastructure also included administrative coordination, patient databases for tracking surveillance schedules, and dedicated staffing time to manage appointments (“It would be supported by some sort of improved administration and for it to really work if you have a database of patients and know when things are due.” SP5). Both GPs and specialists reported needing greater funding support from government in the form of billing codes to enable adequate reimbursement (“Without the billing you can't do it because you just lose money by consulting people. You're working for free and the institution suffers as well.” SP4; “Give poor GPs a bit more rebates… I think that will… encourage GPs to do it.” GP13).

## 4 Discussion

Our study supports the use of telehealth for follow‐up care of CRC survivors provided by both specialists and GPs, within a shared care setting. Amongst our sample of GPs and specialists who provided follow‐up care via telehealth, participants indicated that telehealth offers an acceptable alternative to in‐person care, in the context of routine follow‐up care for known patients.

The growing population of cancer survivors requiring follow‐up care, and the high cost and limited workforce associated with the specialist‐led model has necessitated the investigation of alternative approaches. This is particularly relevant in the context of CRC, which is highly prevalent and requires a substantial number of follow‐up visits per patient, typically for 5 years post‐treatment completion. The possibility of shifting a proportion of these clinic visits to telehealth may alleviate some of the time and cost burden of CRC follow‐up care, for both patients and the health care system [22], as well as free up physical resources such as clinic rooms and provide flexibility for HCPs to work remotely [23]. For patients who are well, remote monitoring and telehealth‐based reviews will likely suffice, enabling healthcare providers to focus in‐person care on those with more complex needs. This approach is aligned with evidence suggesting that physical exams often add little value for asymptomatic CRC survivors [24], and as HCPs in the present study indicated, patients presenting with any concerns can be followed‐up with an in‐person consult. This approach also aligns with policies in countries like the UK, which emphasize remote monitoring for CRC survivors [5]. Key factors identified in our study and elsewhere influencing successful telehealth management in cancer and survivors include the familiarity between physician and patient, optimized care coordination, communication (including cultural factors and need for interpreter) and overcoming logistical and technical concerns [13, 25]. The 2021 American Society of Clinical Oncology (ASCO) standards for telehealth in oncology [26] support the importance of these factors, with appropriate patient selection and establishment of the doctor‐patient relationship stated as important considerations prior to establishing contact via telehealth [13, 25].

Importantly, CRC survivors within the SCORE trial expressed a preference for survivorship care via telehealth, except in situations where they perceived a need for a physical examination, and were satisfied with care received via telehealth [27]. Follow‐up appointments in their traditional format often involve short interactions with HCPs and burden patients with extended waiting and travel times, contagion risk, and travel and parking costs, making telehealth an attractive alternative for patients. Furthermore, telehealth may provide viable and sustainable avenues for CRC survivors to meet their wider survivorship care needs, including care for physical and psychosocial effects of cancer and treatment [11]. Integrating telehealth into CRC follow‐up care (where appropriate) may address the growing demand for survivorship care services and holistic care, in a manner that provides patient benefits while optimizing resource allocation.

Despite its advantages, telehealth implementation faces several challenges, which have been highlighted in our study and elsewhere [11, 28]. Firstly, regarding technology, access to video telehealth varies across both hospitals and GP offices, with hospitals often better equipped. Reliable internet connectivity, suitable devices and IT support staff are essential for effective telehealth delivery. Secondly, for telehealth to be viable, it is essential that policy and reimbursement processes adapt to support this as a new care model. These changes are critical to ensure the sustainability of shared care and telehealth. Thirdly, there are logistical challenges with setting up and billing appointments, as well as administering tests and prescriptions, receiving results and sharing information, which require ongoing administrative support.

Additional concerns raised by HCPs in this study indicated that telehealth may not be suitable for patients without access to technology, which may include older adults or those from lower socioeconomic groups [29], or people in regional areas without reliable internet access [30], and for those with language barriers. Evidence indicates disparities in use of telehealth for cancer care according to certain demographic variables, including older adults aged 70 years and older, those living in rural areas, people of color, low SES and less education [31, 32, 33]. Hence while telehealth offers the potential to increase access to care for those living in rural and remote areas, and also reduce financial burden for patients related to travel, parking and time off work to attend appointments, telehealth may also be associated with disparities regarding who is able to access and use the technology, and potential benefits for some groups may not be realised. Barriers such as language differences may be addressed through solutions like three‐way video telehealth with interpreters, or having interpreters physically present with HCPs. However, more work needs to be done to understand differences in telehealth usage amongst underserved populations, and to develop strategies to mitigate inequities in access and use.

### 4.1 Implications (Clinical and Research)

Findings from this study support the integration of telehealth in CRC survivorship care. Adequate IT infrastructure, administrative support, and streamlined processes for ordering tests, sharing results, coordinating interpreters, and billing are needed to improve efficiency, and sustained government reimbursement mechanisms for telehealth are critical to support uptake. Telehealth appears most appropriate for known patients attending routine follow‐up appointments. A hybrid model of care combining in‐person and telehealth consultations, as appropriate, may maximize both the quality of care and efficient use of health resources, allowing face‐to‐face appointments to be prioritized for patients with complex needs, concerning symptoms or results, or situations requiring physical examination or sensitive communication. Based on our study, factors to consider when determining appropriateness for telehealth follow‐up align with three domains regarding (1) patient access and use of technology (i.e. reliable internet access, access to a smart phone or computer with functional camera, microphone and speaker, ability to download and use required software), (2) patient characteristics (i.e. known patient, adequate hearing, adequate English language skills, ability to communicate health concerns, patient preference) and (3) clinical characteristics (i.e. reason for the appointment, no need for physical examination, no concerns regarding recent test results, patient's overall health status). Future research may develop an evidence‐based decision‐making tool for clinical use to determine patients and appointments suitable for telehealth. Future research may also examine equity in telehealth access and use, and develop and assess strategies to mitigate disparities.

### 4.2 Limitations

This study sought perspectives from both oncology specialists and GPs on providing CRC survivorship care as part of the SCORE trial via telehealth in Melbourne, Australia. Specialists were located within public hospitals and as such, their experience may not be generalizable to the private health care setting. As participants provided care within the context of an RCT, and with some support from trial staff, their experiences may not be generalizable to CRC survivorship care more broadly. Interviews were conducted 18–24 months following completion of the SCORE trial. This may have led to difficulty in recalling the care provided to CRC survivors or recalling specific aspects of telehealth as related to CRC survivorship care.

## 5 Conclusion

This study supports the use of telehealth for the provision of survivorship care of CRC survivors, within certain contexts. Both GPs and oncologists found telehealth to be acceptable and appropriate for the provision of routine CRC follow‐up care, depending on the nature of the appointment and the characteristics of the patient. Challenges related to technology, logistics, and reimbursement were raised; addressing these will be essential to support sustained implementation of telehealth for CRC survivorship care. Future research may address decision making regarding telehealth use and understanding and mitigating disparities in telehealth access.

## Conflicts of Interest

The authors declare no conflicts of interest.

## Supporting information


Supporting Information S1

## Data Availability

Research data are not shared.
