# Supplementary material for: Oncologist and General Practitioner Perspectives of Telehealth‐Delivered Colorectal Cancer Survivorship Care
Source: Psychooncology. 2026 May 19;35:e70491. doi: 10.1002/pon.70491 (PMC13185682; doi:10.1002/pon.70491)
Supplement: Supplementary file 1 — Supporting Information S1 [file PON-35-e70491-s001.docx]

**APPENDIX 1: TELEHEALTH INTERVIEW GUIDES: GPs AND SPECIALSTS**

This interview is part of a study exploring models of survivorship care. You have been asked to participate because you had a patient who received follow-up care for colorectal cancer as part of the SCORE trial. For this interview, we are interested in your experience or perceptions of providing survivorship care via telehealth. There are no right or wrong answers; each person’s experience and opinions are important and we are interested to hear what you think so please be as open and honest as you can. I would also like to reassure you that neither you nor your patient will be identifiable from this research. This interview will be audio-recorded and transcribed verbatim, and the transcript of the interview will be de-identified and used for data analysis. Do you have any questions about anything I have said?

We have previously sent you information regarding the study. Can I please confirm that you have received and read this information provide consent to be interviewed today?

Q: Do you remember if you conducted any of your follow-up appointments remotely via phone or video call?

If NO: Skip to Q*.

If YES:

Q: Can you tell me about the types of phone or video call appointments that you had? (Describe the platform used, how it was set up)

Q: What systems or technology did you have to put in place to facilitate seeing patients remotely?

Q: Is telehealth something that you offered prior to COVID?

Q: How did you/others determine patients that were appropriate for telehealth and patients that were not?

Q: Compared to seeing your patient in person for cancer follow-up, how did you find the phone/video consult?

- What was good about it?/What worked well?
- What didn’t work well?/Did you experience any challenges?

Q: Did you feel that you could provide the same level of care remotely that you do when seeing a patient in person? Why/why not?

- Do you have any concerns about providing survivorship care via telehealth?

Q*: Do you plan to offer telehealth to your patients in the future/post-COVID? Why/why not?

- What would help you to offer telehealth to patients in the future?

Q: If telehealth was to become routinely offered in the future, how do you think this could this be supported?

Q: Is there anything else you would like to tell me about telehealth that we haven’t covered?
